# Engineering and Characterization of an Enhanced Fluorescent Protein Voltage Sensor

**DOI:** 10.1371/journal.pone.0000440

**Published:** 2007-05-09

**Authors:** Dimitar Dimitrov, You He, Hiroki Mutoh, Bradley J. Baker, Lawrence Cohen, Walther Akemann, Thomas Knöpfel

**Affiliations:** 1 Laboratory for Neuronal Circuit Dynamics, Brain Science Institute, RIKEN, Saitama, Japan; 2 Department of Cellular and Molecular Physiology, Yale University School of Medicine, New Haven, Connecticut, United States of America; Duke Unviersity, United States of America

## Abstract

**Background:**

Fluorescent proteins have been used to generate a variety of biosensors to optically monitor biological phenomena in living cells. Among this class of genetically encoded biosensors, reporters for membrane potential have been a particular challenge. The use of presently known voltage sensor proteins is limited by incorrect subcellular localization and small or absent voltage responses in mammalian cells.

**Results:**

Here we report on a fluorescent protein voltage sensor with superior targeting to the mammalian plasma membrane and high responsiveness to membrane potential signaling in excitable cells.

**Conclusions and Significance:**

This biosensor, which we termed VSFP2.1, is likely to lead to new methods of monitoring electrically active cells with cell type specificity, non-invasively and in large numbers, simultaneously.

## Introduction

Previous prototypic fluorescent protein voltage sensors were developed by molecular fusion of a GFP-based fluorescent protein to voltage-gated ion channels or components thereof [1]–[4]. The first prototype, FlaSh, was obtained by inserting GFP in the C-terminus of the *Drosophila* Shaker potassium channel [1]. Another prototype, SPARC, is based on the insertion of GFP into a skeletal muscle sodium channel [4]. Concomitantly, our laboratory explored a different design principle that exploits the voltage-dependent conformational changes of an isolated voltage sensor domain [2], [3]. The prototype based on this design principle, VSFP1, is composed of the voltage sensor domain from the Kv2.1 potassium channel fused to a pair of cyan and yellow fluorescent proteins (CFP and YFP) [2]. In VSFP1, fluorescence resonance energy transfer (FRET) between CFP and YFP changes as a function of membrane potential. Unfortunately, none of the previous fluorescent protein voltage sensors showed satisfactory response characteristics when expressed in mammalian cells, mainly because of poor targeting to the plasma membrane [5].

The detailed mechanism of membrane targeting of Kv potassium channels is poorly understood [6]. There is, however, evidence that interaction between channel subunits is an obligate step for their correct folding and insertion into the plasma membrane [7], [8]. Furthermore, partial folding and subunit interactions may be required to mask ER retentions signals [9]. In addition, Kv channels may undergo cycles of plasma membrane trafficking and internalization, a process that may require features of the natural conformation that is lost following truncation and/or GFP insertion.

Recently, a self-contained voltage sensing domain was identified in the non-ion channel protein Ci-VSP (*Ciona intestinalis*-Voltage-Sensor-containing Phosphatase) [10]. We speculated that the subcellular targeting of the isolated voltage sensing domain of Ci-VSP would be less dependent on the interactions with other membrane proteins and therefore exhibit efficient plasma membrane targeting. Furthermore, as a heterologous protein, it may not be subject to regulatory processes that control plasma membrane expression in mammalian cells.

## Results and Discussion

**Figure 1:**
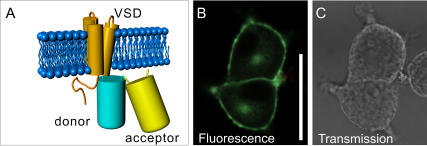
Design and plasma membrane expression of VSFP2s. A: A pair of CFP (donor) and YFP (acceptor) is attached to the 4-transmembrane-voltage-sensing domain (VSD) of Ci-VSP. B, C: Confocal fluorescence (B) and transmission images (C) of PC12 cells transfected with VSFP2D. Note the targeting of the fluorescent protein to the plasma membrane. Scale bar is 30 μm.

To explore this idea we fused a pair of FPs at different distances from the predicted fourth transmembrane segment of Ci-VSP, yielding VSFP2A through D (Figure S1). The pair of FPs consisted of a C-terminally truncated CFP (Cerulean) [11] and a YFP (Citrine) [12] with a one amino acid (S) linker [2]. Consistent with our expectation, all four VSFP2 variants (A through D) displayed bright fluorescence and clear targeting to the plasma membrane of PC12 cells. (Fig. 1A, Figure S2). Clear targeting to the plasma membrane was also observed in HEK 293 cells and primary hippocampal neurons (data not shown).

**Figure 2:**
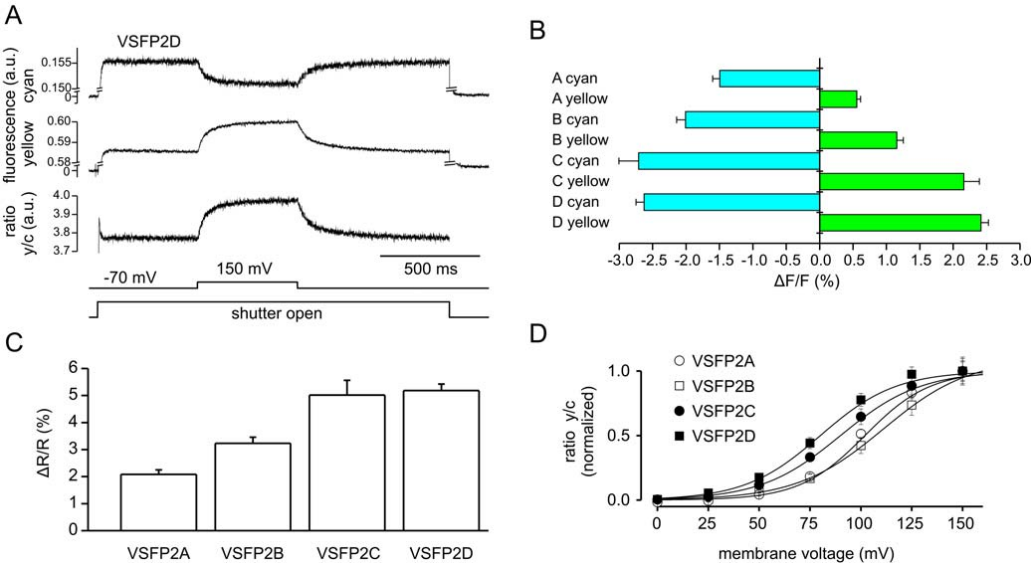
Fluorescence signals induced by membrane depolarization in PC12 cells. A: Sample traces of cyan fluorescence, yellow fluorescence and the ratio of yellow/cyan fluorescence (average of 27 traces). Lower traces indicate times of shutter opening and membrane depolarization from −70 mV to 150 mV. B: Average changes in cyan fluorescence and yellow fluorescence induced by depolarization to 150 mV. Labels A–D indicate VSFP2A (11 cells), VSFP2B (7cells), VSFP2C (7cells), VSFP2D (7 cells). C: Average changes in the ratio of yellow and cyan fluorescence induced by depolarization to 150 mV. (D) Ratio of yellow/cyan fluorescence versus test membrane voltage. Lines are Boltzmann fits. Bars in B–D are SEM.

The relationship between cyan and yellow fluorescence and membrane potential was investigated in voltage-clamped PC12 cells transfected with VSFP2s. All of these initial VSFP2 variants were voltage sensitive when tested with depolarizing voltage pulses from a holding potential of −70 mV to 150 mV. This large depolarization was chosen because it covers the voltage dependency of Ci-VSP gating currents [10]. Voltage steps (duration 500 ms) resulted in a decrease of cyan emission (460–500 nm) and an increase of yellow emission (>515 nm) in VSFP2A through VSFP2D (Fig. 2). Both CFP and YFP signals were smallest in the VSFP2A construct, intermediate in VSFP2B and largest in VSFP2C and VSFP2D (Fig. 2B). Accordingly, the change in the ratio between YFP and CFP fluorescence was smallest in VSFP2A and largest in VSFP2C and VSFP2D (Fig. 2C). The fluorescence-voltage relationship was characterized for membrane voltages ranging from 0 mV to 150 mV (Fig. 2D). ΔR/R values were normalized to the response obtained at 150 mV and plotted as a function of step voltage. Data for each variant were fitted with a Boltzmann equation, yielding half maximal responses (V_1/2_ values) between at +60 to +100 mV (Fig. 2D). ΔR/R slope values ranged from 3.9% to 8%/100 mV and on/off time constants from ∼40 ms to ∼100 ms (Table 1).

**Table 1:** Summary of VSFP2 response properties

Variant recording temperature	VSFP2A	VSFP2B	VSFP2C	VSFP2D	VSFP2.1	
	22°C	22°C	22°C	22°C	22°C	35°C
V_1/2_ (mV)	102	111	91	80	−71	−70
ΔR/R/100 mV (at V_1/2_)	3.9%	4.5%	8.0%	5.9%	6.5%	8.6%
on time constant (ms)	110 (at 150 mV)	109 (at 150 mV)	60 (at 150 mV)	58 (at 150 mV)	71 (at 40 mV)	15 (at 40 mV)
off time constant (ms)	41 (at 150 mV)	71 (at 150 mV)	61 (at 150 mV)	89 (at 150 mV)	96 (at 40 mV)	75 (at 40 mV)

**Figure 3:**
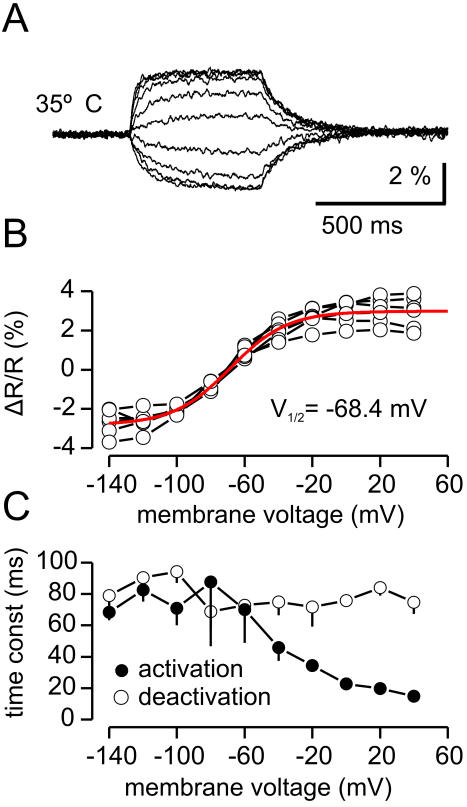
Characteristics of VSFP2.1. Response-voltage relationship and kinetics of VSFP2.1 at 35°C. (A) Ratio of yellow/cyan fluorescence during a family of 500 ms voltage steps from a holding potential of −70 mV to test potentials of −140 mV to +40 mV (20 mV increments). Traces are grand averages over average responses from 6 cells. (B) Ratio of yellow/cyan fluorescence versus test membrane voltage. Connected symbols are data from individual cells. Red line is Boltzmann fit with V_1/2_ value of −68.4 mV. (C) Voltage dependence of on and off time constants.

The response-voltage relationship of VSFP2A-D with V_1/2_ values far above the physiological range of membrane potential fluctuations in electrically active cells make the VSFP2A-D variants of limited use as probes for electrophysiological events. We therefore performed mutational alterations of the positive charges in the putative S4 segment of Ci-VSP with the aim of shifting the voltage dependency to a more physiological region. Exchange of the positively charged arginine at position 217 (R217Q) resulted in a protein that we termed VSFP2.1, which exhibited a V_1/2_ value of ∼−70 mV both at 22°C and at 35°C (Fig. 3). At 22°C on and off kinetics were similar to VSFP2A-D (Table 1). At 35°C the on response was considerably faster (Fig. 3A, C). The functional properties of VSFP2.1 were independently confirmed in HEK 293 cells and primary hippocampal neurons in the laboratory of Larry Cohen at Yale University (using the methods described in [5]; data not shown).

**Figure 4:**
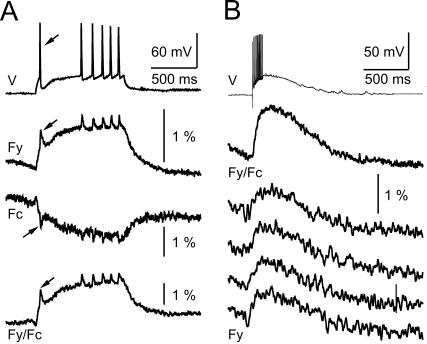
VSFP2.1 responds to physiological neuronal membrane signals. PC12 cells expressing VSFP2.1 were voltage clamped with a voltage trace obtained from a current-clamped mouse olfactory bulb mitral cell. The mitral cell was stimulated to generate a series of action potentials by intracellular injection of a current pulse (A) or by electrical stimulation of the olfactory nerve (B). Traces in (A) are averages of 50 sweeps, upper traces in (B) are the average of 90 sweep and the lower four traces in (B) are single sweeps. Traces show membrane potential (V), yellow fluorescence (F_y_), cyan fluorescence (F_c_) and the ratio of yellow and cyan fluorescence (F_y_/F_c_). Fluorescence signals were digitally low pass filtered (0.2 kHz) and were not corrected for dye bleaching. Recordings were done at 35°C.

The response-voltage relationship with a V_1/2_ value close to the resting potential of neuronal membranes and a relatively fast kinetics makes VSFP2.1 a candidate for optical measurements of neuronal activity such as large synaptic potentials, isolated action potentials or trains of action potentials, and bistabilities in resting membrane potential. To test this prediction, PC12 cells were voltage clamped with membrane potential signals obtained from mouse mitral cells [13]–[15]. For the signal used in Fig. 4A, the mitral cell was stimulated by injection of a current pulse through a patch clamp electrode to generate a series of action potentials. VSFP2.1 could clearly monitor individual action potential events in PC12 cells (arrows in Fig. 4A) as well as the underlying membrane depolarization. As expected from the response kinetics of VSFP2.1, the optical readout of the fast action potentials was reduced relative to the slower components of the membrane potential change. This phenomenon was also clearly seen when using the signal from a mitral cell to a single shock stimulation of the olfactory nerve (Fig 4B). This response consisted of a burst of fast action potentials on top of a slow synaptic potential. The response of VSFP2.1 mainly reported the slow synaptic potential. Importantly, the responses shown in figure 4B could be resolved in single sweeps.

Because of its excellent membrane targeting, reasonably large voltage dependent signal, and relatively fast response time at 35°C, VFSP2.1 represents a major advance in the engineering of fluorescent voltage sensor proteins as monitors of neuron and cardiac activity [16]. Furthermore, expression of VFSP2.1 under cell-specific promoters would allow addressing specific cellular populations within heterogeneous cell assemblies [17], [18].

## Materials and Methods

### Construction of VSFP2x

The cDNA coding sequence for Ci-VSP [10] was a kind gift from Dr. Y. Okamura (NIPS, Okasaki, Japan). High-fidelity PCR (PfuTurbo polymerase; Stratagene, La Jolla, CA, USA) was used for the amplification of the coding sequence and for the introduction of suitable restriction endonuclease sites. For the generation of the four cDNA fragments encoding for the voltage sensor domain of Ci-VSP, a sense primer containing a Kozak consensus sequence flanked by a 5′ NheI site: 5′-ATTA**GCTAGC**GCCACCATGGAGGGATTCGACG GTTCA-3′, and a set of antisense primers containing a NotI site: 5′-ATGCATGA**GCGGCCGC**ATTGTTGATG GGAATAAAATATTC-3′, 5′-AATAGAAT**GCGGCCGC**ATGAAGCCTTCATTTGTTG AT-3′, 5′-TATGTATT**GCGGCCGC**ACTGTGATATTGTTCTTCTGCTTGA-3′, and 5′-TATTTACT**GCGGCCGC**ATCGACGCTTGTTCTGTGATATTGT-3′ were used. The Cerulean (kind gift from Dr. Piston, Vanderbilt University, USA) coding sequence was amplified using the following primer pairs: sense primer with a NotI site: 5′- CAGTCCT**GCGGCCGC**ATGGTGAGCAAGGGCGAGGAGCTG -3′ and antisense primer with a BamHI site: 5′- TCA**GGATCC**GAGAGTGATCCCGGCGGCGGTCACG AACTC -3′. The Citrine (kind gift from Dr. Griesbeck, MPI Martinsried, Germany) coding sequence was amplified using sense primer with a BamHI site: 5′- TGAGCCCG**GGATCC**ATGGTGAGCAAGGGCGAGGAGCTGTTCACC -3′ and the antisense primer containing an EcoRI site: 5′- TCAGCGGCCGCATTA**GAATTC**CTT GTACAGCTCGTCCATGCCGAGAGTGATCCCGGC -3′. PCR fragments encoding for Cerulean and Citrine were digested at their flanking restriction sites and ligated into the NotI/EcoRI sites of the pcDNA3.1(−) vector (Invitrogen, Carlsbad, CA) yielding pcDNA3.1(−)Cer-Cit. The PCR products encoding for the voltage sensor domain of Ci-VSP were ligated at the NheI-NotI sites of pcDNA3.1(−)Cer-Cit. The resulting constructs were named VSFP2A trough VSFP2D, with A to D indicating the increasing lengths of the linker between the voltage sensor domain and the Cerulean-Citrine fluorescent protein pair. For the introduction of the mutations, we used the QuickChangeTM Site-Directed Mutagenesis System (Stratagene, La Jolla, CA) method with PfuTurbo polymerase (Stratagene, La Jolla, CA) and DpnI restriction endonuclease (New England Biolabs, Ipswich, MA) enzymes [19]. All constructs were confirmed by sequence analysis.

### Cell culture and transfection

PC12 cells were grown in DMEM supplemented with 5% fetal calf serum and 10% horse serum. Cells were grown on poly-D-lysine-coated coverslips, transfected (Lipofectamine™ 2000, Invitrogen) with VSFP2 expression plasmids one day after plating, and used for functional studies 34–72 hours after transfection. Confocal images (Fig. 2) were obtained with a Nikon confocal laser scanning microscope (C1si/FN1, Nikon, Tokyo, Japan).

### Electrophysiology and optical imaging

A coverslip with transfected PC12 cells was placed into a recording chamber mounted on the stage of an inverted microscope (Eclipse TE-2000 U, Nikon). The PC12 cells were recorded in whole-cell patch-clamp configuration using an Axopatch 200B patch-clamp amplifier (Axon Instruments, Sunyvale, CA, USA). Glass pipettes (3.5–5 Mohm) were pulled from borosilicate glass using a two-stage vertical puller (PP-830, Narishige, Tokyo, Japan). The pipette solution contained (in mM): K-aspartate 120, NaCl 4, MgCl_2_ 4, CaCl_2_ 1, EGTA 10, Na_2_ATP 3, and HEPES 5 at pH 7.2. External solution contained (in mM): NaCl 150, KCl 4, CaCl_2_ 2, MgCl_2_ 1, Glucose 5, and HEPES 5. Clampex software (Axon Instruments) was used to synchronize application of voltage command pulses and fluorescence excitation. Emission light was directed via a beam splitter (DCLP505), and emission filters (LP515 and D480/40) to two photomultipliers (Viewfinder, T.I.L.L. Photonics, Martinsried, Germany) to measure the yellow and the cyan fluorescence intensities. Excitation light (440 nm) was passed through a band pass filter and a dichroic mirror (D440/20 and DCLP 455nm).

### Data analysis

The fluorescence and electrophysiological signals were analyzed using Clampfit (Axon Instruments) and Origin software (OriginLab, Northhampton, MA, USA). Photobleaching (typically less than 0.1%/s) was corrected by subtraction of a linear fit of the bleaching curve. Fluorescence transients were fitted with a single exponential function of the form
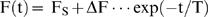



On and off time constants were given by τ, baseline fluorescence levels (F) were given by F_s_ −ΔF for positive ΔF and by F_s_ for negative ΔF and used to calculate ΔF/F (relative change in cyan, ΔF_c_/F_c_, and yellow fluorescence, ΔF_y_/F_y_) values (see Fig. 2A). The relative change in the ratio between yellow and cyan fluorescence (ΔR/R) was calculated as
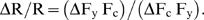



It should be noted that this expression is independent of the relative efficacies of sampling cyan and yellow fluorescence (e.g. of photomultiplier gain). Response-voltage curves were fitted with a Boltzmann equation of the form
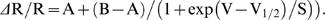



V_1/2_ is the voltage of half maximal response and the slope of ΔR/R at V1/2 was calculated as (A–B)/4s. No attempts were made to correct for background fluorescence (e.g. cellular autofluorescence) and therefore, reported ΔF/F values are lower estimates. The values are presented as mean±SEM.

## Supporting Information

Figure S1 Alignment of the fourth transmembrane segment of Shaker, KvAP, Kv1.2, and Kv2.1 potassium channels with Ci-VSP. For Ci-VSP, two possible alignments are shown. The dotted box represents assignment of the predicted intramembrane portions of the S4 segment (Cuello et al., 2004).
(2.28 MB TIF)

Figure S2 Expression and plasma membrane targeting of VSFP2s. Confocal fluorescence (A1 trough D1) and transmission images (A2 through D2) of PC12 cells transfected with VSFP2A (A1, A2), VSFP2B (B1, B2), VSFP2C (C1, C2), VSFP2D (D1, D2). Scale bar is 30 μM.
(9.99 MB TIF)
