# Supplementary figures and images for: Engineering and Characterization of an Enhanced Fluorescent Protein Voltage Sensor
Source: PLoS One. 2007 May 9;2(5):e440. doi: 10.1371/journal.pone.0000440 (PMC1857823; doi:10.1371/journal.pone.0000440)

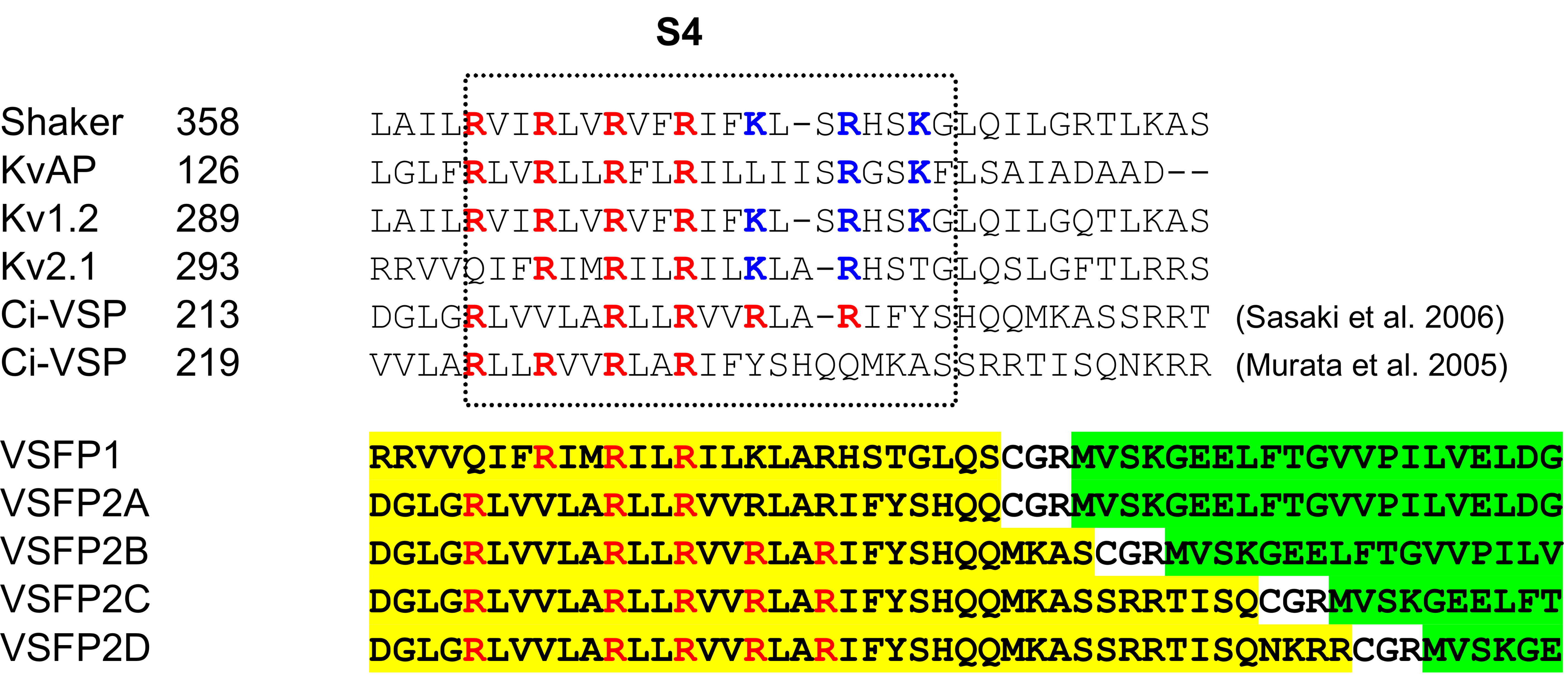

Supplement: Figure S1 — Alignment of the fourth transmembrane segment of Shaker, KvAP, Kv1.2, and Kv2.1 potassium channels with Ci-VSP. For Ci-VSP, two possible alignments are shown. The dotted box represents assignment of the predicted intramembrane portions of the S4 segment (Cuello et al., 2004). (2.28 MB TIF) [file pone.0000440.s001.tif]

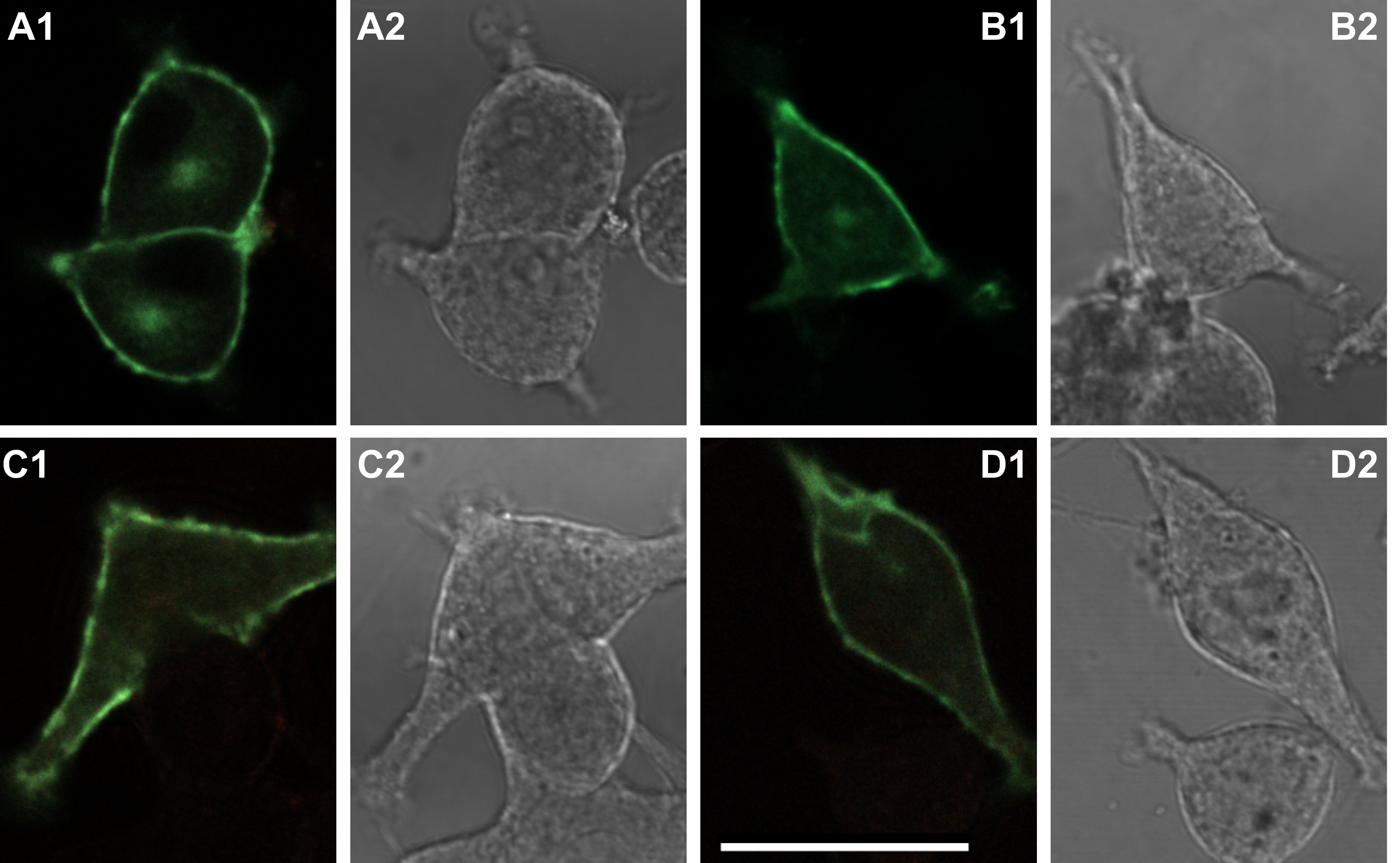

Supplement: Figure S2 — Expression and plasma membrane targeting of VSFP2s. Confocal fluorescence (A1 trough D1) and transmission images (A2 through D2) of PC12 cells transfected with VSFP2A (A1, A2), VSFP2B (B1, B2), VSFP2C (C1, C2), VSFP2D (D1, D2). Scale bar is 30 μM. (9.99 MB TIF) [file pone.0000440.s002.tif]
